# Artificial intelligence in maxillofacial trauma: expert ally or unreliable assistant?

**DOI:** 10.4317/medoral.27229

**Published:** 2025-08-16

**Authors:** Nelli Agbulut, Meric Unlu

**Affiliations:** 1 ORCID: 0000-0001-9860-5494. DMD. Specialist. Assistant Professor. Istanbul Medipol University, School of Dentistry, Department of Oral and Maxillofacial Surgery, Istanbul, Turkey; 2 ORCID: 0009-0003-7366-9995. DMD. Doctorate student. Istanbul Medipol University, School of Dentistry, Department of Oral and Maxillofacial Surgery, Istanbul, Turkey

## Abstract

**Background:**

Large language models (LLMs), such as ChatGPT, have demonstrated potential in synthesizing complex clinical information, yet concerns persist regarding their accuracy and reliability in specialized domains. The rationale of this study is to address a gap in the literature by evaluating ChatGPT-4o’s capabilities and limitations in terms of accuracy and reliability on oral and maxillofacial traumatology.

**Material and Methods:**

A total of 188 oral and maxillofacial trauma-related questions were selected from a comprehensive resource. Thirty questions were randomly chosen and submitted to ChatGPT-4o resetting to “new chat” mode every repetition to eliminate potential memory bias. Accuracy was scored using a 3-point Likert scale. Reliability was assessed with weighted kappa (κ) and Intraclass Correlation Coefficient (ICC), and internal consistency was evaluated using both Cronbach's alpha (α) and McDonald’s omega (ω).

**Results:**

The accuracy rates for comprehensive and adequate responses were calculated as 38% (95% CI: 32.5% - 43.5%) and 58% (95% CI: 52.1% - 63.3%), respectively. Weighted kappa (κ = 0.469) and ICC (0.503) indicated moderate reliability. Internal consistency metrics revealed excellent and good reliability, respectively (α = 0.904, ω = 0.860).

**Conclusions:**

ChatGPT-4o demonstrated promising results as an adjunct tool in providing supplementary educational content, verifying critical information, and supporting the decision-making processes in oral and maxillofacial traumatology. Current limitations warrant further research. Future enhancements in LLMs and prompt engineering may assist in the optimization of their clinical applicability and alignment with evidence-based standards.

** Key words:**Oral surgery, maxillofacial trauma, artificial intelligence, large language models, performance, clinical decision making.

## Introduction

Artificial intelligence (AI) has brought groundbreaking advancements across numerous medical specialties and maxillofacial trauma is no exception. Managing complex injuries in this domain demands precision and timely decision-making. Recent attention has been drawn to large language models (LLMs) for their potential to support diagnosis and treatment planning (1). The reliability of AI tools in clinical decision-making is an increasingly pressing issue, particularly in specialized fields such as maxillofacial trauma. While LLMs have demonstrated promise in processing and synthesizing complex clinical information, concerns about their accuracy and consistency persist. Discrepancies in AI-generated responses may lead to variations in diagnostic interpretations and treatment recommendations, particularly in intricate cases. To address these challenges, integrating AI into clinical workflows requires thorough validation and an evaluation of the dependency on AI-generated insights to safeguard patient outcomes (2,3).

The rapid advancements in AI have reshaped industries, revolutionized knowledge dissemination, and propelled scientific innovation. Among these developments, OpenAI's ChatGPT stands out as a transformative language model capable of producing human-like text. Launched in 2022, ChatGPT is grounded in OpenAI’s pioneering efforts in natural language processing. Trained on extensive datasets, ChatGPT demonstrates an ability to analyze patterns, synthesize knowledge, and generate contextually relevant responses across a wide range of domains (4,5).

The evolution of ChatGPT, culminating in its latest version during the time of this study, ChatGPT-4o, exemplifies the swift pace of AI innovation. The “o” in “4o” symbolizes “omni,” reflecting the model's ambition to provide universally applicable knowledge. Despite initial skepticism about its reliability, ChatGPT has gained widespread recognition for its versatility across sectors such as healthcare, education, and engineering. While research highlights its potential to complement human expertise, ongoing debates emphasize its limitations and the need for careful evaluation of its applications.

Although traditional educational tools like clinical guidelines and textbooks remain critical, the potential of AI tools such as ChatGPT to supplement healthcare providers as a reliable source of information remains underexplored (6). The integration of AI tools into medical decision-making processes and medical education is rapidly expanding, particularly in specialized fields such as oral and maxillofacial traumatology, where making timely and accurate decisions significantly impact patient outcomes. Thus, the rationale of this study is to address a gap in the literature by evaluating ChatGPT-4o’s capabilities and limitations in terms of precision and dependability on oral and maxillofacial traumatology - a subspecialty that has not been investigated in this perspective. Consequently, this study seeks to answer the following question: How accurately and reliably does ChatGPT-4o respond to oral and maxillofacial trauma-related questions?

## Material and Methods

This study utilized the Oral and Maxillofacial Surgery (OMFS) Secrets question-bank textbook which is curated and edited by a team of expert oral and maxillofacial surgeons in teaching, research, and clinical practice, thus representing a comprehensive resource in oral and maxillofacial surgery (7). This particular question bank was chosen due to its proven effectiveness in replicating the format and content of real examination questions with a high degree of fidelity.

196 practice questions related to oral and maxillofacial trauma surgery including dentoalveolar injuries, mandibular trauma, zygomaticomaxillary complex and orbital fractures, midfacial fractures including nasal, nasoethmoidal, and LeFort fractures, frontal sinus fractures, and soft tissue facial injuries underwent an independent review by two examiners (N.A. and M.U.) to ensure appropriateness for the study. Eight questions either involving clinical images, photographs, or questions related to incidence, prevalence or anatomy were excluded from consideration after the screening process. Subsequently, 188 questions were deemed fit for inclusion. 30 questions out of 188 were randomly selected using www.random.org for further analysis (Supplement 1).

The selected questions were introduced into ChatGPT-4o using an independent new account. Each question was submitted individually in a separate session to ensure independence of responses. Given the probabilistic nature of ChatGPT, which may yield variable responses to identical inputs, ten repetitions were performed for each question. The “new chat” mode was reset before every repetition to eliminate potential memory bias (8).

To enhance the specificity and clinical relevance of the responses, the following tailored prompt was employed: “Imagine that you are a professor of oral and maxillofacial surgery with a fellowship on maxillofacial trauma surgery. Please answer the following questions accurately and directly as if you are talking to an esteemed colleague in the oral and maxillofacial surgery field, without rambling or creative answers. Give details and when asked for complications, consider post-operative complications as well: *Question*”.

All responses were archived in a Word document (Microsoft, Redmond, Washington, USA), the accuracy for each answer was subsequently analyzed and the data were noted in an Excel spreadsheet (Microsoft, Redmond, Washington, USA).

According to the answer provided for each question in the textbook, ChatGPT-4o-generated responses were assessed using a 3-point Likert scale as follows: A score of 1 indicated that the response was either inaccurate or inadequate meaning that it was addressing some aspects of the question but leaving significant parts missing or incomplete. A score of 2 was considered adequate, meaning the response addressed all aspects of the question and provided the minimum amount of information required to be deemed complete. Finally, a score of 3 was deemed comprehensive, as it addressed all aspects of the question and provided additional information or context beyond what was expected. This method allowed the authors to systematically evaluate the thoroughness and completeness of the responses (9). Any discrepancies were resolved by the attending oral and maxillofacial surgeon (N.A.).

This study was conducted in adherence to the principles outlined in the Declaration of Helsinki. An Ethics Committee or Institutional Review Board approval was not required due to the use of publicly available data and the nature of the study not involving any human subjects or previously collected human material.

Statistical analysis was carried out using JASP version 0.19.2 statistical software (University of Amsterdam, Amsterdam, The Netherlands). All analyses were performed along with their corresponding 95% confidence intervals (CI). The absolute (n) and relative (%) frequency of scores — 1 (inaccurate or inadequate), 2 (adequate), and 3 (comprehensive)—were calculated.

Test-retest reliability for the consistency of responses across ten repetitions was evaluated using linear weighted kappa (κ) and Intraclass Correlation Coefficient (ICC). Cronbach's alpha (α) was used to assess the internal consistency of ChatGPT-4o’s responses to 30 questions at each repetition. To overcome Cronbach's alpha's bias in estimating internal consistency for Likert-type scales due to its assumption of tau-equivalence, McDonald's omega (ω), as a more accurate measure of reliability, was also employed (10). Calculations regarding weighted kappa, ICC, and Cronbach’s α and ω were interpreted using guidelines suggested within the literature, which are displayed on Table 1 (11-13).

Detailed analyses on accuracy were assessed depending on how well the responses aligned with the textbook answers which was considered the expected standard (ground truth) (7). Accuracy among the generated answers was identified as the proportion of questions yielding an answer with a score of 3 (comprehensive) or 2 (adequate) and was calculated separately and cumulatively (scores of both 2 and 3), along with a 95% CI using the Wald binomial method.

## Results

The absolute (n) and relative (%) frequency of scores for each question considering the Likert-scale (i.e. 1 - inaccurate or inadequate, 2 - adequate, or 3 - comprehensive) are listed at Supplement 2.

Weighted kappa (κ) was calculated as 0.469 suggesting that each response across ten repetition is moderately reliable (Table 2). Overall weighted kappa values ranged from -0.157 to 0.986, with the majority representing moderate to substantial reliability. Confidence intervals highlighted variability in agreement strength.

A value of 0.503 was calculated for ICC, similarly, suggesting moderately reliable (consistent) responses (Table 3). The 95% CI for ICC ranged from 0.368 to 0.661, with the lower bound suggesting poor reliability and the upper bound falling within the moderate range. This wide range suggests variability in the consistency of ratings. Although an overall moderate agreement is evident, variability as shown by the wide CI indicates some inconsistency.

Cronbach's alpha and McDonald’s omega were calculated as 0.904 and 0.860, which indicate excellent and good internal consistency, respectively (Table 4).

The accuracy rates for comprehensive and adequate responses were calculated as 38% (95% CI: 32.5% - 43.5%) and 58% (95% CI: 52.1% - 63.3%), respectively. The overall cumulative accuracy rate incorporating both comprehensive and adequate responses was 96% (95% CI: 93.4% - 98.0%).

## Discussion

To this date various studies have examined ChatGPT’s accuracy and reliability regarding answers pertaining to several medical specialties and subspecialties, but to our knowledge, no studies have evaluated it, in this context, regarding oral and maxillofacial traumatology-related questions.

Although accuracy rates reported in similar studies vary greatly, ranging from 35-93.6%, authors believe that the utility of LLMs in medical education and decision support should be emphasized owing to their continued self-refinement and evolution of the AI technology, though certain limitations still warrant discussion (14-17). Research methodologies involving older versions of ChatGPT or the use of multiple-choice question formats may be accounTable for a slightly higher cumulative accuracy rate of 96% reported in this study than the upper range reported in the literature, as well as other studies reporting much lower rates.

Studies employing a three-point Likert scale have revealed that ChatGPT-4o scores worse in terms of response completeness (i.e. comprehensive (score of 3) versus adequate (score of 2)) when evaluated against open-ended questions related to oral and maxillofacial traumatology, compared to questions on multiple specialties, where higher rates of comprehensive responses (53.3% and 71.6%) have been reported as opposed to the findings from this study (38%) (17). The higher rates reported in other studies may result from differences and variations in methodology and diverse evaluation criteria. 

ChatGPT has been shown to provide varying answers to the same question (17). As a result, the consistency of its responses has been assessed by asking the same question multiple times and evaluating response consistency across repetitions using various metrics. Previous research examining ChatGPT’s accuracy and reliability in medical inquiries across specialties including physical therapy and rehabilitation, urology as well as oral surgery have indicated moderate levels of consistency, parallel to the results of this study (17,18). Although wide confidence intervals, reflecting variability in reliability, may stem from the probabilistic nature of LLMs and their sensitivity to prompt phrasing and input context, this issue may benefit from algorithmic improvements or more standardized prompt engineering in near future (19).

Internal consistency has been evaluated with both Cronbach's alpha and McDonald’s omega and a slight discrepancy between these values has been noted. The decision to apply both methods was notably due to Cronbach's alpha’s slight overestimation of reliability and previous research suggesting McDonald’s omega as a more accurate measure of reliability for Likert-type scales (12,13). Findings on internal consistency in this study diverge from those in the literature, where consistency levels have been ranging from questionable to accepTable (20,21). Superior results in terms of internal consistency in this study may be linked to the study’s carefully-crafted prompt which may have helped improve not only the internal consistency but also the accuracy of responses (19).

The rapid evolution of LLMs has introduced various AI systems with potential applications in healthcare, although their efficacy remains under investigation. A recent study evaluating four LLMs in the context of oncological knowledge has reported that Claude 3.5 Sonnet outperformed others, with ChatGPT-4o following closely behind. In contrast, Llama-3 and Gemini 1.5 have demonstrated lower accuracy, suggesting that more advanced LLMs may serve as valuable tools for oncological education and clinical decision-making (22). Furthermore, a comparison of treatment decisions from a multidisciplinary tumor board, ChatGPT, and Gemini has revealed moderate agreement in decisions regarding surgery and radiotherapy, with ChatGPT also showing moderate alignment for chemotherapy decisions, outperforming Gemini overall (23). Another study examining triage referrals has reported that ChatGPT’s suggestions are more aligned with clinical practice than those of Gemini, although Gemini performed slightly better in diagnoses (24). Discrepancies among different LLMs may be attributed to the lack of medical-specific tailoring in these models, as well as variations in the quality of input data. Finally, when compared to Med-Go, an LLM specifically trained for medical use, both ChatGPT-4 and Gemini have been outperformed, highlighting the advantages of specialized fine-tuning of medical expertise in LLM architecture (25).

Despite AI’s growing capabilities, studies continue to emphasize the superiority of hybrid decision-making models that combine AI with human clinicians. Although our study highlights the promising performance of ChatGPT-4o in answering oral and maxillofacial trauma-related questions, its applicability in real-world clinical decision-making remains constrained by several limitations. A recent study has reported that ChatGPT-4 achieved an 80% accuracy rate at deciding a final diagnosis regarding oral and maxillofacial diseases, when provided with standard diagnostic descriptions, compared to accuracy rates of 87%, 24%, and 17% for dental surgeons specialized in oral medicine and pathology, general dental surgeons, and dental students, respectively. These findings suggest that while AI does not surpass human expertise, it performs comparably well and may serve as a valuable adjunct, particularly for non-specialists. Notably, when ChatGPT-4 has been provided with descriptions derived from study participants, its accuracy of 80% declines to 62%. Despite still outperforming non-specialists, this decline emphasizes the limitations of AI-driven decision support tools, which, while demonstrating high accuracy in structured question-answering tasks, may struggle with context-dependent clinical decision-making (15). These discrepancies suggest that AI should be viewed as a complementary tool, rather than a replacement for clinicians, by providing rapid and broad differential diagnoses, which must then be refined by specialists.

Research on AI alone and hybrid human-AI decision-making models suggests that AI alone may remain inadequate for managing complex and unpredicTable clinical scenarios. Rather than functioning as an autonomous decision-maker AI serves better an assistive tool, that when integrated with human expertise has been shown to enhance clinical outcomes beyond the capabilities of AI or human clinicians alone (26). Thus, AI-enhanced decision-making frameworks have the potential to improve accuracy, efficiency, and overall clinical judgment by improving AI’s power with human expertise (27). Recent research including a study group of two distinct ethnicities and genders has revealed that physicians are more likely to adjust their clinical decisions with the assistance of ChatGPT-4, resulting in enhanced clinical accuracy. AI-driven clinical decision-making may enhance medical practice notably without being influenced by demographic biases, thus additionally ensuring equiTable care across diverse patient populations (28). However, these systems also introduce challenges, such as automation bias, where an over-reliance on AI-generated recommendations without thoroughly assessing their validity may impair the decision-making process (29).

Even when AI replaces clinical decision-making, humans will still play a role in creating and inputting data, which may have an impact on data integrity. On the other hand, introducing low-quality data into advanced algorithms may result in misdiagnoses or mistreatment, when not properly checked by humans. Additionally, since AI systems will be trained primarily on data from developed English-speaking countries, potential exacerbation of global health inequalities rather than a reduction may be ineviTable. The lack of controls and checks in these systems also increases the potential for new risks. While advocates argue that AI's integration is ineviTable, it is essential not to blindly embrace a future where AI replaces human input, as the autonomy of patients and clinicians is essential (30).

Several limitations of this study must be acknowledged. First, the exclusion of questions involving anatomy, incidence and prevalence, as well as visual aids such as clinical or radiological images, restricts the generalizability of the findings and may potentially influence the study’s findings by narrowing the scope of question complexity. Future research should incorporate a more diverse range of question types to provide a comprehensive evaluation of ChatGPT-4o's assessment. Second, the probabilistic nature of LLMs and their sensitivity to prompt phrasing may have led to variability in response consistency. This may be mitigated by refining and optimizing AI-human collaboration frameworks, exploring advanced prompt engineering techniques, or model-fine tuning. Finally, the assessment on purely textbook knowledge, without real-world clinical integration, may limit the applicability of these findings to actual clinical practice. Future research on LLMs should prioritize incorporating dynamic updating capabilities to ensure alignment with the latest evidence-based practices to enhance their reliability and clinical applicability.

This study highlights the promising potential of ChatGPT-4o in healthcare, particularly in its ability to provide supplementary educational content, verify critical information, and support the decision-making processes. While the performance metrics of ChatGPT-4o are encouraging, addressing its limitations remains essential for optimizing its role as an effective and reliable tool in healthcare. The findings of this study reveal that ChatGPT-4o’s application in oral and maxillofacial trauma care may be better suited as an adjunct tool rather than a standalone decision-maker alongside human practitioners. Its ability to provide reasonably accurate information with rapid retrieval may be especially beneficial in trauma care when timely access to reliable information is crucial. To maximize its utility, continued efforts must focus on refining its capabilities and mitigating its shortcomings, ensuring its alignment with the up-to-date standards of clinical practice and ethical considerations.

## Figures and Tables

**Table 1:** Interpretation of statistical metrics.

	Calculation	Interpretation
Weighted kappa (κ) (11)	Less than 0	Poor agreement/reliability
	0.01 - 0.20	Slight agreement/reliability
	0.21 - 0.40	Fair agreement/reliability
	0.41 - 0.60	Moderate agreement/reliability
	0.61 - 0.80	Substantial agreement/reliability
	0.81 - 1	Almost perfect agreement/reliability
Intraclass Correlation Coefficient (ICC) (12)	Less than 0.50	Poor reliability
	0.50 - 0.75	Moderate reliability
	0.75 - 0.90	Good reliability
	0.90 - 1	Excellent reliability
Cronbach's alpha (α) (13)	Less than 0.5	Unacceptable
	0.5 - 0.6	Poor internal consistency
	0.6 - 0.7	Questionable internal consistency
	0.7 - 0.8	Acceptable internal consistency
	0.8 - 0.9	Good internal consistency
	Greater than 0.9	Excellent internal consistency
McDonald's omega (ω) (13)	0.6 - 0.7	Questionable internal consistency
	0.7 - 0.8	Acceptable internal consistency
	0.8 - 0.9	Good internal consistency
	Greater than 0.9	Excellent internal consistency

**Table 2:** Weighted kappa.

Ratings	kappa	SE	95% CI	
			Lower	Upper
Weighted kappa (linear)	0.469	0.145	0.185	0.753

**Table 3:** Intraclass Correlation.

Type	Point Estimate	Lower 95% CI	Upper 95% CI
ICC3,1	0.503	0.368	0.661

Note. 30 subjects and 10 measurements.

**Table 4:** Bayesian Scale Reliability Statistics.

Coefficient	Posterior mean	95% CI	
		Lower	Upper
McDonald's ω	0.860	0.792	0.924
Cronbach's α	0.904	0.852	0.950
